# Effect of anthocyanin-rich blackberry juice on endoplasmic reticulum stress in streptozotocin-induced diabetic rats

**DOI:** 10.1007/s11356-023-27827-z

**Published:** 2023-06-07

**Authors:** Sallam K. Tony, Mohamed SH. Hassan, Hamadi A. Ismail, Gamal F. Abd El-Naem, Hanaa S. S. Gazwi

**Affiliations:** Department of Agricultural Chemistry, Faculty of Agriculture, Minia University, El-Minia, Egypt

**Keywords:** Blackberry juice, Endoplasmic reticulum stress, IL-6, ATF4

## Abstract

This study investigates blackberry juice’s effects on glucose metabolism, oxidative stress, inflammation, and endoplasmic reticulum stress (ER) in streptozotocin (STZ)-induced diabetic rats. Fifty Wistar rats were distributed to five groups randomly of ten rats each: Normal control, diabetic control, 9 mL/kg body weight (b.wt) blackberry juice only, blackberry juice plus diabetes, and 500 mg/kg b.wt metformin plus diabetes. A single intraperitoneal injection of 50 mg/kg b.wt STZ induced diabetes in the rats. This animal study continued for 56 days after the confirmation of diabetes. The levels of liver function and renal function, as well as insulin, glucose-6-phosphatase, glucokinase, and malondialdehyde (MDA) levels, and the activities of catalase (CAT) and superoxide dismutase (SOD), were determined. Additionally, interleukin-6 (IL-6) levels, tumor necrosis factor-alpha (TNF-α), and activated transcription factor 4 (ATF4) expressions were examined in the liver homogenate of rats. Furthermore, the liver tissues were utilized for histopathological examination. The results showed that blackberry juice prevented drastic loss of body weight and reduced food consumption in diabetic rats. Additionally, the levels of blood glucose, total protein, aspartate aminotransferase (AST), albumin, alanine aminotransferase (ALT), uric acid, creatinine, and urea improved after the administration of blackberry juice in diabetic rats. Blackberry juice significantly increased glucose metabolism and antioxidant status while reducing ER stress and inflammation in diabetic rats. Moreover, blackberry juice improved glucose metabolism by increasing insulin levels and improving the dysregulated activities of glucose-metabolizing enzymes. The microstructure of liver tissues in diabetic rats was also improved with blackberry juice treatment. Therefore, blackberry juice has the potential to alleviate diabetes in rats and could be considered as a functional food for people with diabetes.

## Introduction

Diabetes mellitus is a chronic metabolic disorder characterized by high blood glucose levels resulting from insulin resistance or impaired insulin secretion, or both (Katsarou et al. 2017). Type 2 diabetes (T2D) is the most common form of diabetes, accounting for approximately 90% of all diabetes cases (American Diabetes Association 2020). The global prevalence of diabetes was 9.3% in 2019 and is expected to increase to 10.2% by 2030 and 10.9% by 2045 (Saeedi et al. 2019).

One of the key organelles involved in T2D is the endoplasmic reticulum (ER), where protein synthesis and folding occur, making it a crucial component of cellular function. The unfolded protein response (UPR) is a pro-survival process that is triggered by disruptions in protein production homeostasis and ER folding capacity. However, ER stress, which is often linked to apoptosis and inflammation, results from the persistent activation of the UPR by factors such as excessive lipid load, hyperglycemia, oxidative stress, and excessive Ca2 + release from ER storage (Ozcan et al. 2004). Pancreatic β-cells, among other specialized cells, are responsible for producing, folding, and processing insulin (Oyadomari et al. 2002).

Blood glucose levels are regulated by enzymes that catalyze reactions in the gluconeogenic and glycolytic pathways, such as phosphofructokinase, hexokinase, and glucose-6-phosphatase (Petersen et al. 2017). Effective normal control of blood glucose levels is critical for treating and preventing diabetes and its consequences.

Diabetes complications can be reduced through lifestyle changes, such as dietary changes (Gregg et al. 2014). Nutrition also plays a significant role in the development or reduction of metabolic defects, and can therefore be considered a non-pharmacological supplement to the treatment of acute illnesses (Sears and Ricordi 2012). Blackberries are rich in phenolic chemicals, including ellagitannins and anthocyanins (Kaume et al. 2012). The black mulberry, or *Morus nigra* L., is the healthiest type of mulberry due to its high concentration of flavonoids, anthocyanins, and other phenols (Ercisli and Orhan 2007). These compounds have various health benefits, including protection against cardiovascular disease, inflammation, diabetes, cancer, and age-associated neurodegenerative illnesses (Kaume et al. 2012). These health benefits are attributed to the compounds' ability to reduce oxidative stress, which is the leading cause of disease (Finkel 2011).

Berries can be considered a source of exogenous antioxidants that can be obtained through diet and may help treat diabetes. The aim of this study was to evaluate the ability of anthocyanin-rich blackberry juice to mitigate inflammation, glucose metabolism, antioxidant status, and ER stress in diabetic rats.

## Methods and materials

### Preparing blackberry fruit juice

Fresh blackberry fruits were obtained from a garden in Minia, Egypt, washed, and homogenized to prepare the juice. For the preparation of the juice, a commercial Philips blender from China was used to blend the fruits. After blending, the resulting juice was diluted with distilled water in a 1:1 ratio and filtered through a Büchner funnel to remove any solid pieces, resulting in a clear liquid. The dosage of blackberry juice used in the experiment was based on previous studies by Ismail et al. (2014) and Siriwoharn et al. (2004), which recommended a dose of 9 mL/kg body weight.

### Determination of bioactive compounds in anthocyanin-rich blackberry fruit juice

The procedure involves weighing 5 g of dried blackberry juice, soaking it in 25 mL of ethanol for a day, filtering it through Whatman No.1 filter paper and the solvent evaporated in vacuo at 50 °C. The resulting sample was then analyzed using Bioactive Compounds, HLPC, and GC/MS technology.

The total phenolic content (TPC) was determined using the Folin-Ciocalteu test (Maurya and Singh 2010) and was expressed as the amount of gallic acid equivalent per g of dry material. The total flavonoid concentration was measured using the procedure described in Jia et al. (1999) and expressed as quercetin equivalent per g of dry material. The total concentration of anthocyanins were determined using the method described by Ranganna (1977). The results are presented in milligrams of cyanidin 3-glucoside per 100 g.

### Identification of anthocyanins in blackberry by HPLC

The HPLC analysis was done following the method described by Drust and Wrolstad (2001). It was carried out using an Agilent HPLC-LC1620A series model from Santa Clara, CA, USA, which was equipped with a degasser, an automatic injector for the autosampler, a high-pressure pump, and a UV/Visible detector with the ability to detect at various wavelengths. The chromatograms were monitored at 521 nm.

### GC/MS analysis

The chromatography–mass spectrometry (GC–MS) analysis of blackberry juice was performed following a published procedure (Soraya et al. 2022). A thermal scientific trace GC Ultra/ISQ single Quadrupole MS, TG-5MS fused silica capillary column was utilized for the GC/MS analysis at the National Research Center, Dokki, Giza (30 m, 0.251 mm, and 0.1 mm film thickness). For GC/MS detection at a constant flow rate of 1 ml/min, an electron ionization system with 70 eV ionization energy and helium gas as the carrier gas was used. The injector and MS transfer line were kept at a constant temperature of 280 °C. To investigate the quantification of all the found components, a percent relative peak area was utilized. To establish a preliminary identification, the retention durations and mass spectra of the compounds were compared to those of the National Institute of Standards and Technology (NIST), WILLY library data from the GC/MS instrument.

### Biological experiment protocol

Fifty male albino Wistar rats, weighing approximately 150 ± 20 g and 2 months old, were obtained from the Animal House et al. Nahda University’s Faculty of Pharmacy in Egypt. The rats were housed in polypropylene cages with husk bedding and maintained under a 12-h light–dark cycle during the study. They were fed a commercial pelleted diet and had access to water and libitum. The experiment was carried out according to the Ethics Committee for the care and use of animals, microorganisms and living cell cultures in education and scientific research at Faculty of Agriculture, Minia University (MU/FA/006/12/22).

### Experimental design

The rats were distributed into five groups comprising ten animals each.

*Normal control group*: Animals received distilled water (10 mL/kg b.wt) by an oral tube for 56 days).

*Blackberry juice group*: Animals received blackberry juice at a dosage of 9 mL /kg b.wt by an oral tube for 56 days (Ismail et al. 2014).

*Diabetic group*: Animals treated STZ/50 mg/kg b.wt (Samarghandian et al. 2017).

*Diabetic group* + *blackberry juice*: Animals were treated with STZ followed by blackberry juice (9 mL g/kg b.wt) by an oral tube for 56 days.

*Diabetic group* + *metformin group*: Animals were treated with STZ followed by metformin (500 mg/kg/b.wt) by an oral tube for 56 days (Dimo et al. 2007).

Rats were given STZ in the form of a single intraperitoneal (i.p.) injection (50 mg/kg b.wt) in citrate buffer (pH 4.5) to induce diabetes, rats in the normal control group were given an identical but non-diabetic dose of buffer (Furman 2015). Those with sustained fasting blood glucose levels > 250 mg/dL 3 days after STZ injection were diagnosed with diabetes. Blackberry fruit juice was first administered 48 h following the injection of STZ.

The study lasted 56 days. The animals’ daily food amounts were recorded, and weights were recorded every week during that period. Food consumption and feeding efficiency ratio (%) were calculated. Blood glucose levels were measured on weekly tail blood samples during fasting by glucose reagent strips (Accu chek®, Roche Diagnostics, Indianapolis, USA). At the end of the trial, the rats were fasted overnight, anesthetized with ketamine (40 mg/kg body weight), and injected intraperitoneally (i.p.) with xylazine (5 mg/kg body weight) before being euthanized by cervical dislocation. Samples of blood were taken from the retro-orbital plexus in tubes. The tubes do not contain anticoagulants to separate the blood sample by centrifugation at 3000 g for 15 min and stored at − 20 °C till used in analyzing the samples biochemically. Small hepatic samples were placed in a 10% formalin solution to conduct the histopathological test. Other liver tissue samples were homogenized and utilized to evaluate MDA and CAT concentrations.

### Biochemical assays

The serum total protein levels, aspartate aminotransferase (AST), albumin, alanine aminotransferase (ALT), uric acid, creatinine, urea, and direct bilirubin were evaluated by a commercial enzymatic kit following the instructions of the manufacturing company. During the difference between total protein and albumin, globulin was calculated. Serum total lipids (TL), total cholesterol (TC), triglycerides (TG), as well as high-density lipoprotein cholesterol (HDL-C), were assayed with a commercially available assay kit from Bio- Diagnostic Co., Egypt. Serum low-density lipoprotein cholesterol (LDL-c) and very low-density lipoprotein cholesterol (VLDL-c) were estimated following Lee and Nieman (1996) and Castelli et al. (1977), respectively.

### Antioxidant biomarkers

Homogenization of liver tissue samples was performed in phosphate buffer at ice cold temperatures (pH7.4), and centrifugation at 3000 × g for 15 min was employed to separate the supernatant from homogenates, which was then used for biochemical analysis. The assay of malondialdehyde (MDA), superoxide dismutase (SOD), catalase activity (CAT), and glutathione peroxidase (GPx) was done in homogenate liver according to Aebi (1984), Ohkawa et al. (1979), Nishikimi et al. (1972), and Rotruck et al. (1973), in liver homogenate, respectively.

### Glucose metabolism analysis

Insulin level, glucose-6-phosphatase activity, glucokinase activity, and hepatic glycogen content in the liver homogenate were estimated using the methods of Harper (1959), Brandstrup et al. (1957), and Roe and Dailey (1966), respectively.

### Inflammatory biomarkers

ELISA kit was used to measure the concentration of IL-6 and TNF-α in liver homogenate following the manufacturer’s instructions using a test reagent kit (CUSABIO Company, 7505 Fannin St. Ste 610–312, Houston, TX 77054, USA).

### Gene expression analysis

Following the manufacturer’s protocol, TRIzol Reagent (15,596,026, Life Technologies, USA) was used to carry out the total purification of RNA from blood samples. Briefly, Invitrogen™ TRIzol™ Reagent is an available reagent for isolating high-quality total RNA (in addition to proteins and DNA) from the cells and tissues of bacteria, yeast, plant, animal, or human in an hour. It is a monophasic solution of guanidine isothiocyanate, phenol, as well as other proprietary elements that enable isolating various small- or large-sized RNA types. It enables sequential precipitation of proteins, DNA, and RNA, using one single sample (Chomczynski 1993). After sample homogenization with TRIzol™ Reagent, chloroform can be provided, and the homogenate can separate into a transparent upper aqueous layer (with RNA), an interphone, and a red lower organic layer (with DNA and proteins). RNA is precipitated from the aqueous layer with isopropanol. DNA is precipitated from the interphase/organic layer with ethanol. Protein is precipitated from the phenol-ethanol supernatant by isopropanol precipitation. After washing the precipitated RNAs, DNAs, or proteins to get rid of impurities, they can be resuspended. A reverse-transcription of 1 μg of total RNA into single-stranded complementary DNA was possible by employing QuantiTects Reverse Transcription Kit (Qiagen, USA) using a random primer hexamer in a two-step RT-PCR reaction, where any genomic DNA (gDNA) impurity was removed by gDNA Wipeout buffer. Total cDNA (30 ng) was used as a template for amplifying using a certain primers pair (Table 1) employed at a concentration of 300 nM. The samples were exposed to real-time PCR in duplicate, and then the duplicates’ mean values were employed to be analyzed subsequently. GADPH was used as a reference gene. Rotor-Gene Q gathered data automatically and conducted the analysis of the threshold cycle (Ct) value standardized to an average Ct value of the housekeeping genes (∆Ct). Moreover, the relative expression of each representative was estimated as 2^−∆∆ct^.

**Table 1:** Probed primers

Target gene	Probe	References
ATF4	5′-GTTGGTCAGTGCCTCAGACA-3′ 5′-CATTCGAAACAGAGCATCGA-3′	Chen et al. (2014)
GADPH	5′-AGAAGG CTGGGGCTCATTTG-3′ 5′-AGGGGCCAT CCACAGTCTTC-3′	Jing-Jing et al. (2013)

### Histopathological examination

Liver specimens were fixed in the solution of formalin (10%) before being rinsed in water, dehydrated in ascending grades of alcohol, and then cleaned in Xylene. Paraffin slices (5 µm) were cut from the specimens and stained with hematoxylin–eosin (H&E) to conduct a normal histopathological trial following Bancroft and Gamble’s method (2008). The assay of histopathological abnormalities in the livers was done, and scores (0–3) were taken by calculating the lesions’ percentage in 5 randomly inspected microscopic fields per animal. The percentage grades were: 0% = lack of change, 1% = slight change, 2% = moderate, and 3% = significant change. Changes below 30% were considered minor, 30–50% were deemed reasonable, and > 50% were considered severe (Ahmed et al. 2019).

### Statistical analyses

Statistical analyses were carried out by SPSS 21.0. For group comparison, the one-way analysis of variance (ANOVA) was run before Tukey’s post hoc test. Additionally, *P* value < 0.05 was set to denote statistical significance. The data expression took the form of means ± standard errors (SE).

## Results

### Total flavonoids, total polyphenol concentration, and anthocyanin profile

The blackberry was reported to have total anthocyanin content (TAC) of about 312.9 mg cyanidin 3-glucoside/100 g DW, total flavonoids (TFs) of about 36.7 ± 1.49 mg QE / g DW, and total polyphenol concentration (TPC) of about 28.80 ± 1.29 mg GAE/  g DW.

**Fig. 1:**
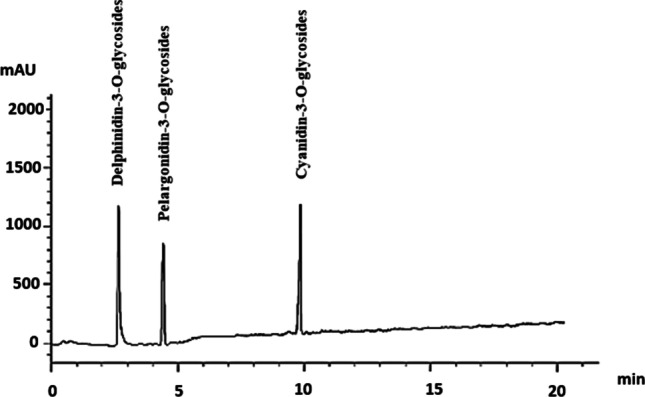
HPLC chromatogram of blackberry anthocyanins at 521 nm

**Fig. 2:**
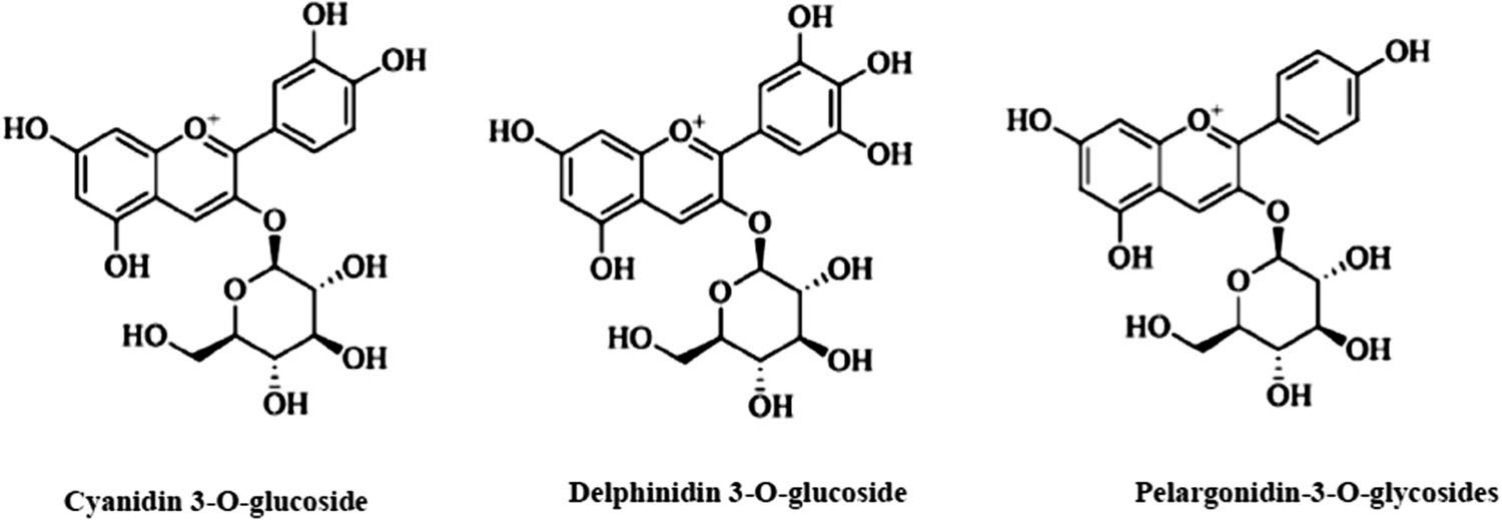
The structures of anthocyanins in blackberry juice

Figure 1 shows the chromatographic analysis of blackberry anthocyanins at a wavelength of 521 nm. The analysis revealed the presence of three types of anthocyanins in blackberries: delphinidin-3-O-glycosides, pelargonidin-3-O-glycosides, and cyanidin-3-O-glycosides (Figs. 1 and 2). The most prevalent anthocyanins were delphinidin-3-O-glycosides (10.12 μg/mg DW), as shown in Table 2.

**Table 2:** Concentration of anthocyanins (µg/mg) identified by HPLC analysis in blackberry juice

RT	Compound	Concentration (μg/mg)
2.8	Delphinidin-3-O-glycosides	10.12
4.4	Pelargonidin-3-O-glycosides	6.55
10.0	Cyanidin-3-O-glycosides	9.85

### GC/MS analysis of blackberry juice

**Fig. 3:**
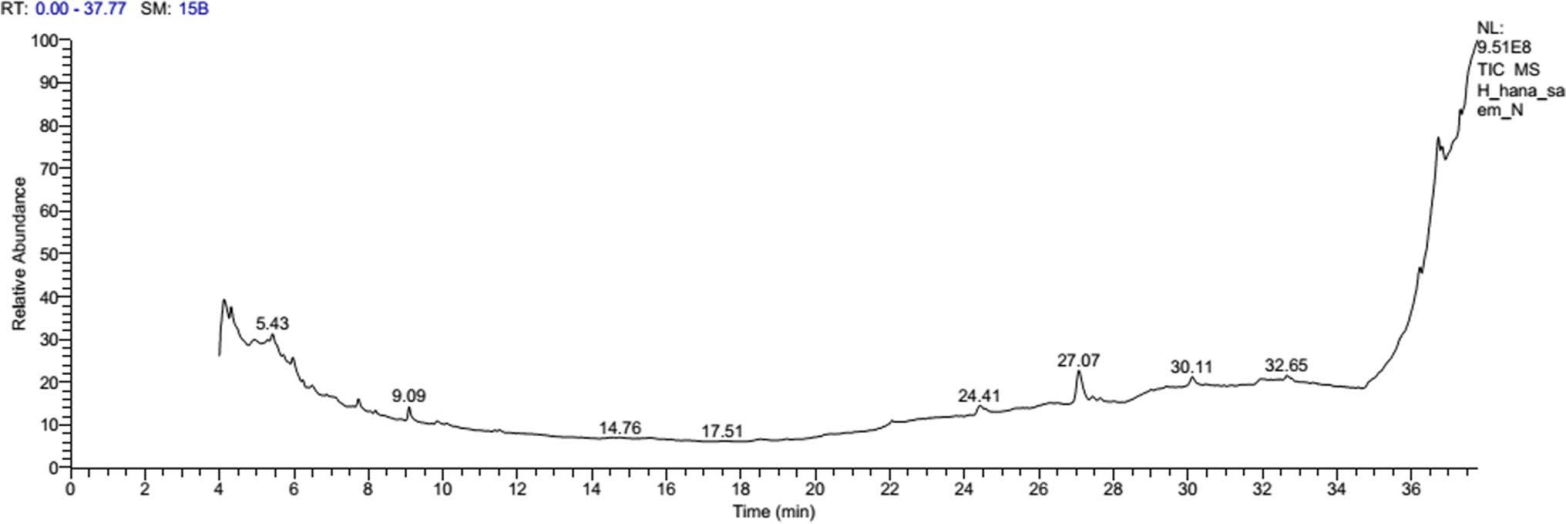
GC–MS chromatogram of blackberry juice

The compounds present in the blackberry juice were identified by GC–MS analysis (Fig. 3). Table 3 lists the active compounds in the blackberry juice, together with their concentration, MW, molecular formula, and retention time (RT). In blackberry juice, 10 chemicals were discovered. The major compounds in the blackberry juice were as follows: 9-borabicyclo-9-borabicyclo-9-borabicyclo-9-bor [3.3.1] nonane,9-[(2-pyridyl) amino]- (22.77%), benzenediamine, n,à,à-trimethyl (5.44%), 3,4,5,6-tetrahydroxy-2-oxo-hexanoic acid (9.32%), 1-deoxy-d-mannitol (3.42%), 2-nonyloxirane (3.16%), 2,4-pyridinedicarboxylic acid, dimethyl ester (3.75%), oxacyclotetradecane-2,11-dione, 13-methyl (11.57%), 11-octadecenoic acid, methyl ester (14.78%), 6H-1,2,5-oxadiazolo[3,4-E]indole-6,8a-diol, 4,5,5a,7,8,8a-hexahydro-, 3-oxide (6.07%), and Z,Z-4,16-octadecadien-1-ol acetate (3.54%).

**Table 3:** GC–MS analysis of blackberry juice

No	RT	Name of the compound	MF	Area %	MW	Activity*
1	4.12	9-Borabicyclo [3.3.1] nonane,9-[(2-pyridyl) amino]-	C_13_H_19_BN_2_	22.7	314	Antioxidant, antimicrobial, anticancer activity, anti-inflammatory, anthelmintic
2	4.31	Benzeneethanamine, n, à,à-trimethyl	C_6_H_9_FO_4_	5.44	164	No report activity
3	5.43	3,4,5,6-Tetrahydroxy-2-oxo-hexanoic acid	C_6_H_10_O7	9.32	194	No report activity
4	5.97	1-Deoxy-d-mannitol	C_6_H_14_O_5_	3.42	166	Antibacterial, antipyretic
5	7.73	2-Nonyloxirane	C_11_H_22_O	3.16	170	No report activity
6	9.09	2,4-Pyridinedicarboxylic acid, dimethyl ester	C_9_H_9_NO_4_	3.75	195	No report activity
7	27.06	Oxacyclotetradecane-2,11-dione, 13-methyl	C_14_H_24_O_3_	11.57	240	Biological activities
8	36.72	11-Octadecenoic acid, methyl ester	C_19_H_36_O_2_	14.78	296	Antibacterial, antifungal, antioxidant, decreased blood cholesterol
9	36.81	6H-1,2,5-Oxadiazolo[3,4-E]indole-6,8a-diol, 4,5,5a,7,8,8a-hexahydro-, 3-oxide	C_8_H_11_N_3_O_3_	6.07	213	No report activity
10	37.30	Z,Z-3,15-Octadecadien-1-ol acetate	C_20_H_36_O_2_	3.54	308	No report activity

^*^Dr. Duke’s Phytochemical and Ethnobotanical Databases

**Fig. 4:**
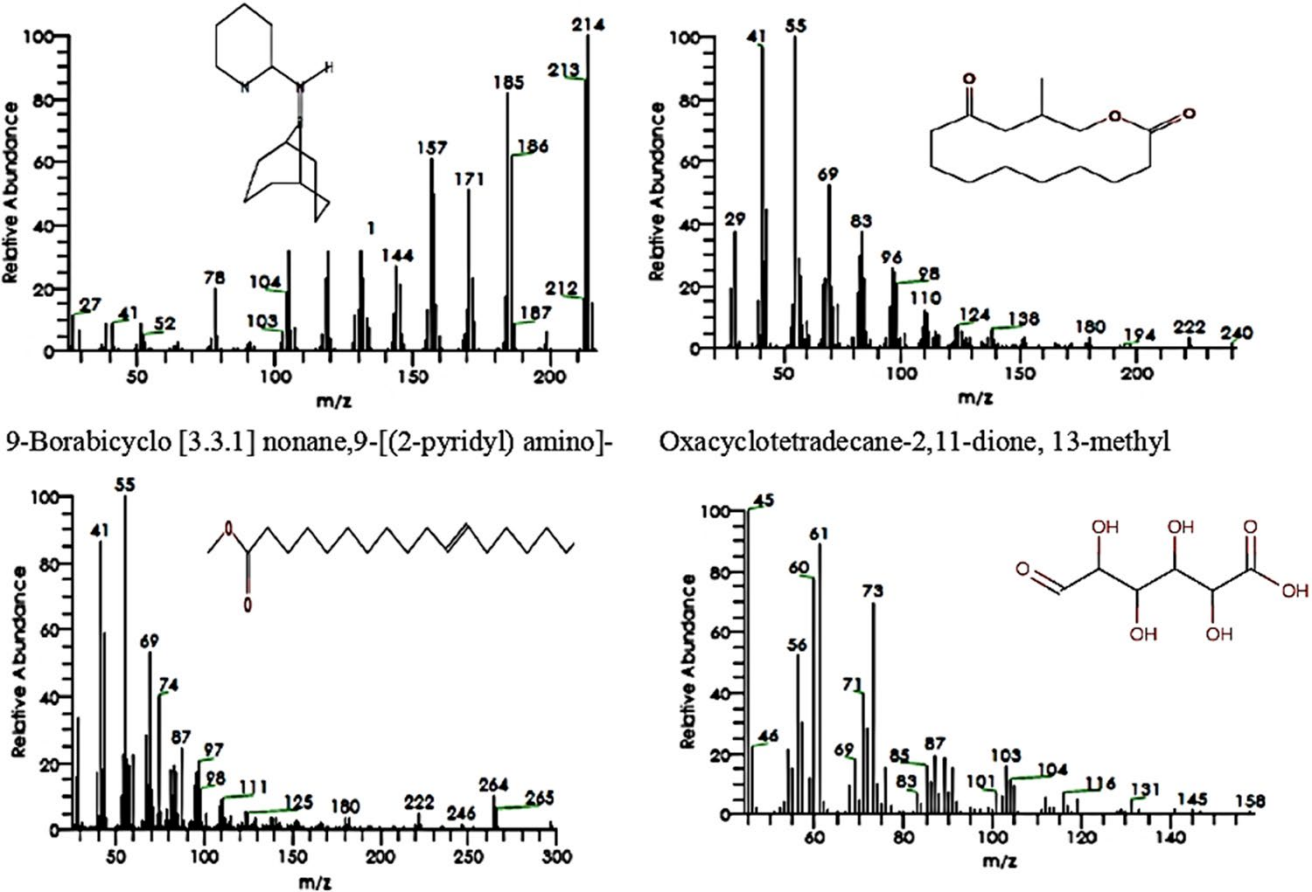
Mass spectra and molecular structures of major components in blackberry juice

Figure 4 shows the mass spectra and structures of 9-borabicyclo [3.3.1] nonane,9-[(2-pyridyl) amino]- (22.77%), 11-octadecenoic acid, methyl ester (14.78%), oxacyclotetradecane-2,11-dione, 13-methyl (11.57%), and 3,4,5,6-tetrahydroxy-2-oxo-hexanoic acid (9.32%), respectively. The GC–MS analysis of blackberry juice yielded the following phytocomponents and their biological activity (Table 3).

### Changes in body weight

Table 4 illustrates the final weight (g), gained weight of the body (g), daily gained weight of the body (g), daily feed consumption (g), and feeding efficiency ratio (%) of the normal control and diabetic rats. There was a significant reduction (*p* < 0.05) in the final weight, gained weight of the body, daily gained weight of the body, feeding efficiency ratio (%), and considerable elevation (*p* < 0.05) in daily feed intake in the diabetic control group compared to the normal control group.

**Table 4:** Effect of blackberry juice on final weight (g), body weight gain (g), daily feed intake (g), daily body weight gain (g), and feed efficiency ratio (%) in diabetic rats (mean ± SE)

Groups	Final weight (g)	Body weight gain (g)	Daily body weight gain (g)	Daily feed intake (g)	Feed efficiency ratio (%)
Normal control	276.85 ± 19.25	88.43 ± 4.41	1.58 ± 0.75	19.03 ± 2.95	8.63 ± 1.36
Blackberry juice	254.05 ± 8.32	85.17 ± 8.51	1.56 ± 0.33	20.23 ± 2.98	8.05 ± 1.29
Diabetic control	157.10^a^ ± 9.46	25.07^a^ ± 1.17	0.87^a^ ± 0.44	24.41^a^ ± 0.45	3.00^a^ ± 0.31
Diabetic + blackberry juice	218.33^ab^ ± 36.04	44.53^ab^ ± 3.33	1.10^ab^ ± 0.23	22.93^ab^ ± 1.17	4.80^ab^ ± 0.26
Diabetic + metformin	222.20^ab^ ± 7.63	48.33^ab^ ± 8.22	1.16^ab^ ± 0.04	22.93^ab^ ± 1.17	4.62^ab^ ± 0.29

^a^Significance difference from the normal control group at p < 0.05. ^b^ Significance difference from the diabetic control group at the level of *p* < 0.05

When diabetic rats were given blackberry juice, these symptoms were normalized, showing that diabetic control is needed to stop muscle loss (Table 4). Blackberry juice and the medication had comparable results (*p* < 0.05) (Table 4).

### Changes in blood glucose levels

Animals with diabetes had sharply elevated (*p* < 0.05) blood sugar levels in comparison with the normal control group (Table 5). However, after 56 days of treatment, glucose levels considerably dropped in all treated groups in comparison with rats with diabetes (Table 5).

**Table 5:** Effect of blackberry juice on blood glucose level in diabetic rats (mean ± SE)

	Changes of blood glucose (mg/dL)									
Groups		0th day	7th day	14th day	21th day	28th day	35th day	42th day	49th day	56th day
Normal control	82.00 ± 2.74	83.50 ± 2.59	79.50 ± 1.19	88.75 ± 6.65	89.00 ± 11.05	104.75 ± 3.66	101.75 ± 5.20	98.25 ± 4.88	97.75 ± 4.73	98.75 ± 5.07
Blackberry juice	82.25 ± 2.87	82.25 ± 2.87	77.75 ± 1.79	92.50 ± 8.82	92.50 ± 3.50	93.25 ± 4.64	94.00 ± 3.32	93.00 ± 4.10	94.25 ± 1.89	93.25 ± 3.52
Diabetic control	81.00 ±1.95 11.95	318.25^a^ ± 15.34	337.00^a^ ± 22.64	391.75^a^ ± 48.74	405.75^a^ ± 10.54	408.75^a^ ± 67.13	410.75^a^ ± 8.19	426.00^a^ ± 20.95	435.75^a^ ± 15.82	447.50^a^ ± 18.98
Diabetic + blackberry juice	81.00 ± 1.58	436.75^a^ ± 54.64	375.00^a^ ± 43.53	336.50^ab^ ± 68.44	271.00^ab^ ± 99.98	255.00^ab^ ± 56.32	237.75^ab^ ± 99.46	215.00^ab^ ± 92.22	197.25^ab^ ± 61.64	168.25^ab^ ± 17.14
Diabetic + metformin	81.75 ± 2.29	383.00^a^ ± 49.94	343.75^a^ ± 91.97	336.25^ab^ ± 15.19	253.25^ab^ ± 42.71	213.25^ab^ ± 60.41	192.25^ab^ ± 44.85	190.25^ab^ ± 26.55	186.75^ab^ ± 31.15	168.25^ab^ ± 38.96

^a^Significance difference from the normal control group at *p* < 0.05

^b^Significance difference from the diabetic control group at the level of *p* < 0.05

### Changes in liver functions

Table 6 displays the mean values of serum protein, albumin, ALT, and AST for the normal control and experimental groups. STZ-induced diabetic rats revealed a substantial increase (*p* < 0.05) in serum protein, albumin, ALT, and AST compared to the normal control ones.

**Table 6:** Effect of blackberry juice on liver function in diabetic rats (mean ± SE)

Groups	AST (U/mL)	ALT (U/mL)	Protein (mg/dL)	Albumin (mg/dL)
Normal control	24.33 ± 3.84	25.33 ± 1.76	7.14 ± 0.16	4.68 ± 0.09
Blackberry juice	25.33 ± 0.88	26.67 ± 3.53	7.23 ± 0.10	4.95 ± 0.12
Diabetic control	88.67^a^ ± 4.49	92.67^a^ ± 7.69	11.55^a^ ± 0.12	7.77^a^ ± 0.19
Diabetic + blackberry juice	64.67^ab^ ± 1.20	52.67^ab^ ± 3.28	10.17^ab^ ± 0.13	7.16^ab^ ± 0.12
Diabetic + metformin	43.33^ab^ ± 3.18	45.33^ab^ ± 2.40	8.92^ab^ ± 0.10	5.83^ab^ ± 0.07

^a^Significance difference from the normal control group at *p* < 0.05

^b^Significance difference from the diabetic control group at the level of *p* < 0.05

There was a significant improvement of these parameters after ingesting blackberry juice and metformin (Table 6).

### Changes in kidney functions

Table 7 summarizes the impact of blackberry juice on the biochemical markers related to the kidneys. The serum levels of creatinine, uric acid, and urea showed a significant increase in rats with diabetes (*p* < 0.05), indicating that these animals had impaired kidney function.

**Table 7:** Effect of blackberry juice on kidney function in diabetic rats (mean ± SE)

Groups	Urea (mg/dL)	Creatinine (mg/dL)	Uric acid (mg/dL)
Normal control	37.58 ± 1.56	0.86 ± 0.04	5.00 ± 0.29
Blackberry juice	38.42 ± 0.72	0.89 ± 0.03	5.67 ± 0.17
Diabetic control	82.10^a^ ± 0.42	1.83^a^ ± 0.06	9.50^a^ ± 0.76
Diabetic + blackberry juice	62.82^ab^ ± 1.06	1.30^ab^ ± 0.06	7.33^ab^ ± 0.44
Diabetic + metformin	54.11^ab ^± 0.67	1.12^ab^ ± 0.06	6.67^ab^ ± 0.44

^a^Significance difference from the normal control group at *p* < 0.05

^b^Significance difference from the diabetic control group at the level of *p* < 0.05

While there were reductions in the serum levels of creatinine, uric acid, and urea by 23%, 23%, and 29%, respectively, in STZ-diabetic rats treated with blackberries, the reductions were statistically significant (*p* < 0.05) (Table 7). Similarly, metformin reduced the increases in these measures due to diabetes by 34%, 29%, and 39%, respectively, in comparison with the group with diabetes (*p* < 0.05).

### Impact of blackberry juice on glucose metabolism

Table 8 shows no significant differences between the blackberry juice group and the normal control in hepatic glycogen level, insulin level, glucokinase activity, and glucose-6-phosphatase activity after 56 days. The STZ-treated group had a significant increase (*p* > 0.05) in glucose-6-phosphatase activity by about 75.54%. In contrast, the insulin, glucokinase, and glycogen activity were significantly lowered (*p* > 0.05) by approximately 86.48%, 74.38%, and 72.51%, respectively, compared to the normal control group.

**Table 8:** Effect of blackberry juice on glucose metabolism in diabetic rats (mean ± SE)

Groups	Glucose-6-phosphatase (U/g tissue)	Insulin (µg/L)	Glucokinase (U/g protein)	Glycogen (mg/g tissue)
Normal control	13.33 ± 0.26	5.03 ± 0.58	9.25 ± 0.42	61.25 ± 1.07
Blackberry juice	13.50 ± 0.95	4.12 ± 0.11	9.35 ± 0.81	62.08 ± 2.27
Diabetic control	23.40^a^ ± 0.32	0.68^a^ ± 0.05	2.37^a^ ± 0.15	16.84^a^ ± 0.73
Diabetic + blackberry juice	17.50^ab^ ± 0.17	1.63^ab^ ± 0.14	4.64^ab^ ± 0.21	24.45^ab^ ± 1.48
Diabetic + metformin	16.34^ab^ ± 0.26	3.51^ab^ ± 0.28	4.90^ab^ ± 0.13	46.40^ab^ ± 0.89

^a^Significance difference from the normal control group at *p* < 0.05

^b^Significance difference from the diabetic control group at the level of *p* < 0.05

The activity of glucose-6-phosphatase appeared significantly (*p* < 0.05) lower in treatment with blackberry juice and metformin concurrently with STZ. Blackberry decreased the elevation of glucose-6-phosphatase by about 25.21% in rats treated with STZ. Metformin concurrently with STZ significantly (*p* > 0.05) decreased glucose-6-phosphatase activity by about 30.17% compared with the STZ group. Table 8 shows that diabetic rats given blackberry juice for 56 days had significantly (*p* < 0.05) higher levels of insulin level and glycogen level, and glucokinase activity than diabetic control rats. In contrast, no differences were found between the metformin and blackberry juice groups.

### Impact of blackberry juice on the oxidative stress of rats with diabetes

Table 9 illustrates the lack of a significant difference between the blackberry-only treated and the normal control groups in MDA level, SOD, CAT, and GPX activities after 56 days. It shows some markers of the antioxidant status, such as GPX, CAT, and SOD, in the liver homogenate. Also, Table 9 illustrates the levels of lipid peroxidation that indirectly indicate free radical damage.

**Table 9:** Effect of blackberry juice on oxidative enzymes in diabetic rats (mean ± SE)

Groups	SOD (U/mL)	CAT (U/g protein)	MDA (nmol/g)	GPX (U/mL)
Normal control	244.00 ± 22.52	1.03 ± 0.04	2.17 ± 0.16	135.33 ± 8.09
Blackberry juice	242.02 ± 10.97	1.61 ± 0.17	2.25 ± 0.17	144.00 ± 22.51
Diabetic control	54.33^a^ ± 7.22	0.19^a^ ± 0.04	9.59^a^ ± 0.35	27.33^a^ ± 4.63
Diabetic + blackberry juice	143.67 ^ab^ ± 2.03	0.82^ab^ ± 0.07	6.83^ab^ ± 0.31	73.00^ab^ ± 2.89
Diabetic + metformin	142.67^ab^ ± 1.63	0.95^ab^ ± 0.04	4.33^ab^ ± 0.29	101.01^ab^ ± 1.15

^a^Significance difference from the normal control group at *p* < 0.05

^b^Significance difference from the diabetic control group at the level of *p* < 0.05

In the utilized STZ model, reducing the activities of CAT and SOD was clear in the liver tissue of the diabetic group in comparison with the normal control group. The group with diabetes lost 81.55% and 77.73% of their activities of CAT and SOD, respectively, in comparison with the normal control one. Also, the treated with STZ had a significant decrease (*p* < 0.05) in GPX activity in the liver (− 79.80) in comparison to the normal control group. In contrast, the group with diabetes had a high level of MDA, i.e., 341.93 3%, compared to the normal control group.

According to the activities of CAT, SOD, and GPX, blackberry juice improved the antioxidant capacity to moderate levels between the diabetic and the normal control groups. The blackberry juice intake increases the activity to almost that of the normal control group, denoting that the blackberry relieves oxidative stress in rats. Therefore, blackberry juice revealed a significance (*p* < 0.05) in the activities of CAT, SOD, and GPX in the livers (164.44%,331.58%, and 167.11%, respectively) compared with the diabetic group. At the same time, the blackberry juice manages to decrease the lipid peroxidation levels in the liver (− 28.78%) to intermediate stations between the diabetic and the normal control groups.

### Blackberry juice’s impact on the inflammatory markers of rats with diabetes

The findings in Table 10 showed no significant difference between the blackberry juice and the normal control groups in TNF-α and IL-6 activities after 56 days. Meanwhile, the treated with STZ increased significantly (*p* < 0.05) the IL-6 and TNF-α by about 9.3- and 4.8-fold compared with the control normal control group.

**Table 10:** Effect of blackberry juice on the inflammation marker in diabetic rats (mean ± SE)

Groups	IL-6 (pg/mL)	TNF-α (pg/mL)
Normal control	84.00 ± 2.31	190.67 ± 3.18
Blackberry juice	87.67 ± 4.33	193.66 ± 3.17
Diabetic control	785.67^a^ ± 7.12	914.01^a^ ± 13.89
Diabetic + blackberry juice	245.34^ab^ ± 3.18	316.02^ab^ ± 6.93
Diabetic + metformin	103.33^ab^ ± 6.36	250.34^ab^ ± 4.33

^a^Significance difference from the normal control group at *p* < 0.05

^b^Significance difference from the diabetic control group at the level of *p* < 0.05

Blackberry juice significantly (*p* < 0.05) reduced increased hepatic TNF-α and IL-6 by about 65% and 69%, respectively, in rats with diabetes in comparison to only rats with diabetes (Table 10). Similarly, metformin significantly (*p* < 0.05) decreased the levels of hepatic TNF-α and IL-6 by about 84% and 87%, respectively, in rats with diabetes in comparison to only rats with diabetes (Table 10).

### Blackberry juice modulates hepatic ER stress in diabetic rats

**Fig. 5:**
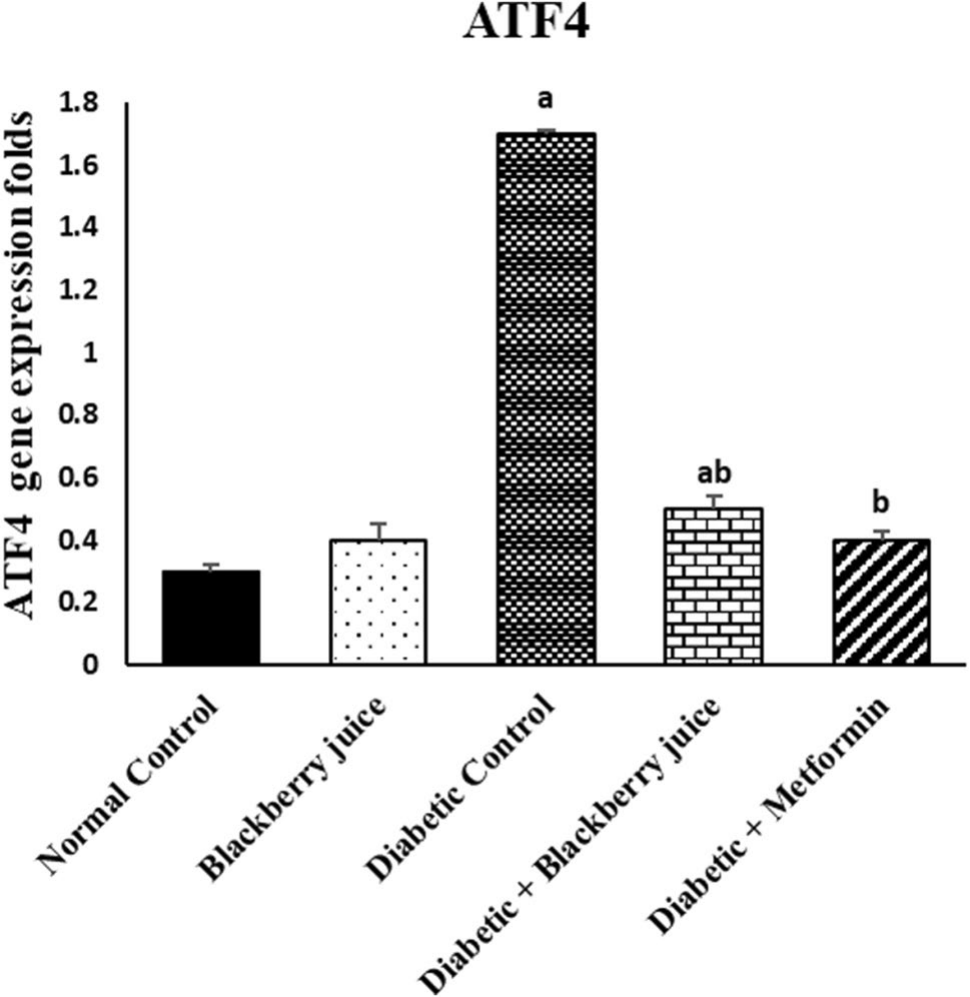
Effect of blackberry juice on ATF4 in diabetic rats (Mean ± SE). ^a^Significance difference from the normal control group at *p* < 0.05. ^b^Significance difference from the diabetic control group at the level of *p* < 0.05

ATF4 expression was (*p* < 0.05) higher significantly in rats with diabetes in comparison with the normal control group (Fig. 5). In contrast, diabetic animals that received blackberry juice were significantly lower (*p* < 0.05) in the expression of ATF4 compared to diabetic animals. Blackberry juice and metformin had similar effects in suppressing ATF4 expression.

### Histopathological examination of the liver

**Fig. 6:**
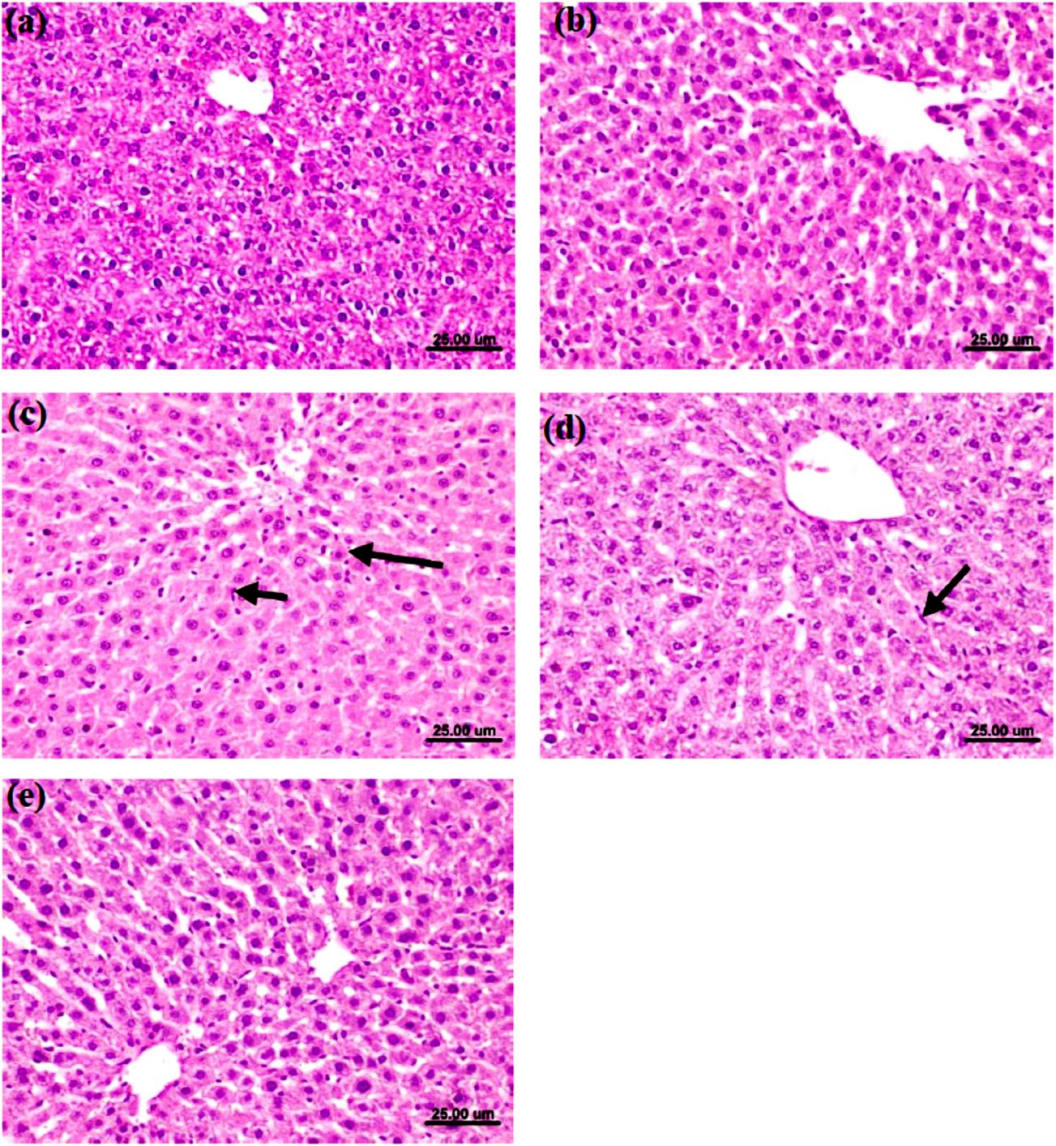
Photomicrographs of the liver of different groups. **a** Photomicrographs of the livers of rats in the normal control group illustrating the normal histoarchitecture of hepatic tissue. **b** Photomicrographs of the livers of rats in the blackberry juice group displaying no histopathological lesions. **c** Photomicrographs of the livers of rats in the diabetic control group displaying the activation of the Kupffer cells and the necrosis of sporadic hepatocytes. **d** Photomicrographs of the livers of rats in the (diabetic + blackberry juice) group displaying slight Kupffer cells activation. **e** Photomicrographs of the livers of rats in the (diabetic + metformin) group 8 displaying the lack of histopathological alterations. (H&E × 400, scale bar 25 μm)

Microscopically, rats’ livers from the normal control group revealed the normal histoarchitecture of hepatic tissue (Fig. 6a and Table 11). Furthermore, the liver of rats from the blackberry juice group exhibited no histopathological lesions (Fig. 6b and Table 11). On the contrary, the diabetic control group rats’ livers displayed activating the Kupffer cells, necrosis of sporadic hepatocytes, hepatocellular vacuolar degeneration, portal edema, and thickening in the wall of the bile duct (Fig. 6c and Table 11).

**Table 11:** The liver’s histopathological lesion score

Histopathological lesion	Normal control	Blackberry juice	Diabetic control	Diabetic + blackberry juice	Diabetic + metformin
Kupffer cells activation	0,0,0,0,0	0,0,0,0,0	3,3,3,2,2	2,2,2,1,1	1,1,0,0,0
Hepatocellular vacuolar degeneration	0,0,0,0,0	0,0,0,0,0	3,3,2,2,2	0,1,0,0,0	0,1,0,0,0
Portal edema	0,0,0,0,0	0,0,0,0,0	1,2,2,1,2	0,0,0,0,0	0,0,0,0,0
Thickening in the wall of bile duct	0,0,0,0,0	0,0,0,0,0	2,2,2,2,3	0,0,0,0,0	0,0,0,0,0

(0) denoted the lack of changes, whereas (1), (2), and (3) showed mild, moderate, and severe changes

Meanwhile, the liver of rats from the diabetic + blackberry juice group described no changes except for slightly activating the Kupffer cells, sporadic hepatocytes necrosis, and slightly vacuolating some hepatocytes (Fig. 6d and Table 11). In contrast, the liver from the diabetic + metformin group displayed no histopathological alterations except for slight vacuolation of some hepatocytes and slight Kupffer cells activation (Fig. 6e and Table 11).

## Discussion

When it comes to functional foods, researchers place more importance on the bioavailability of the nutrient rather than the amount of the nutrient itself in dietary supplements or foods (D’Archivio et al. 2010).

Diabetes metabolic control is influenced by body weight. In rats with diabetes, the final weight was significantly lower than that of the normal control group, likely due to the failure to use glucose for energy, increased use, and decreased protein storage, which depletes body proteins. These symptoms are indicative of type 2 diabetes in humans and animals, resulting from insulin deficiency (Swanston-Flatt et al. 1990). The reduced body weight in diabetic rats compared to normal rats suggests increased protein breakdown (Mishra et al. 2011). Jacobson (2010) found that STZ-induced diabetes leads to weight loss in rodent models because proteins are used for energy when carbohydrates are unavailable. Blackberry juice was found to increase body weight in diabetic rats, highlighting the importance of controlling diabetes to prevent muscle loss. Li et al. (2012) demonstrated that herbs in the diet can improve the feed conversion ratio and increase weight gain by boosting nutrient consumption and gut flora function. Berries contain active compounds such as flavonols, anthocyanins, and ellagitannins, which can improve protein assimilation and digestion (Yilmaz 2019).

The results showed that blood glucose levels increased significantly in infected diabetic rats compared to the normal control group. Compared to diabetic rats, blackberry juice reduced blood glucose levels by 62%. The reduction was statistically insignificant in comparison with the normal control or metformin-treated groups. Ragavan and Krishnakumari (2006) showed that blackberry fruit extract and its anthocyanin-rich fraction could considerably reduce blood glucose levels in rats with diabetes. Basu and Lyons (2012) concluded that various berries had an inhibitory action against α-glucosidase, α-amylase, and pancreatic lipase activities. Furthermore, He and Giusti (2010) reported a competitive attachment of the sodium-glucose co-transporter 1 (SGLT-1) with polyphenols, allowing the suggestion of a reduction in the uptake of glucose in the intestine, improving the hypoglycemic impact. Blackberry juice protected pancreatic beta cells from necrosis and apoptosis by decreasing lipid peroxide activity, enhancing catalase activity leading to a progressive recovery of insulin output, and improved management of hyperglycemia. Blackberry juice and its anthocyanin-rich fraction can have a significant impact on reducing the levels of blood glucose in rats with diabetes, possibly because of potent antioxidant chemicals (Zargar et al.^2014^).

Diabetic rats exhibited a significant increase in serum protein, albumin, ALT, and AST levels compared to the normal control group, indicating potential liver damage and dysfunction due to the metabolic changes associated with diabetes. Diabetes was also related to a significant rise in serum ALT and AST activity, as shown by Yazdi et al. (2019). Ghara et al. (2021) reported that STZ raised both blood glucose and liver enzyme activity (ALT and AST). Hepatocyte injury is detected by increased levels of ALT and AST in the blood (Mansourian et al. 2019). In the blackberry group, the serum levels of AST and ALT decreased by about 27 and 51%, respectively, compared with the diabetic group. This decrease may be due to its antioxidant properties, which protect against diabetes-related liver damage. Ismail et al. (2014) discovered that therapy with strawberry or blackberry alone decreased AST and ALT levels.

In rats with diabetes, impaired glucose metabolism accelerates protein catabolism, leading to an increase in blood urea levels. High blood sugar also causes kidney damage, resulting in elevated levels of creatinine and uric acid as demonstrated in the results of this study. Blackberries reduced serum creatinine, uric acid, and urea levels in diabetic rats. This finding is consistent with the previous report by Hassanalilou et al. (2017) that showed significantly lower serum levels of creatinine, uric acid, and urea in treated rats with diabetes compared with the normal control group after four weeks of administration of the blackberry extract. Abouzed et al. (2020) found that diets high in anthocyanins can reduce the incidence of diabetes and its complications. The present findings corroborate previous research on the putative biological activities of cyanidin 3-glucoside and cyanidin 3-rutinoside (Qin et al. 2018), demonstrating the potential utility of blackberry juice as a neuroprotectant in diabetic animals.

Diabetic rats showed increased glucose-6-phosphatase activity and decreased insulin levels, glucokinase activity, and hepatic glycogen levels compared to the normal control group. Defects in insulin production and action cause hyperglycemia and other metabolic abnormalities associated with T2D through uncontrolled hepatic gluconeogenesis and dyslipidemia due to the dysregulation of fatty acid, triglyceride, and lipoprotein metabolism (Muoio and Newgard 2008). Furthermore, the inability of the β-cells to secrete the amount of insulin required to maintain euglycemia is known to contribute to the development of T2D (Glaser 2007). The decreased insulin secretory capacity of β-cells observed in type 2 diabetes was associated with oxidative stress and inflammation in islet beta cells, causing beta cell death and loss of beta cell mass (Montane et al. 2014). Glucokinase catalyzes a rate-restricting step in the hepatic glucose consumption pathway by phosphorylating glucose to glucose-6-phosphate. Therefore, glucokinase is important in the regulation of blood glucose levels (Agius 2009). Additionally, glucose-6-phosphatase is an important regulator of the gluconeogenesis pathway and affects the development of hyperglycemia (Woerle et al. 2008). Altered enzymes of glucose metabolism were shown in T2D rats, as evidenced by decreased glucokinase activity and increased glucose-6-phosphatase activity (Patel and Goyal 2011). These changes lead to decreased glycogen levels in the liver and hyperglycemia (Ahmed et al. 2012). Our results showed that blackberry juice significantly improved insulin secretion in rats with diabetes, which can result from the effect of therapy on the secretory capacity of β-cells, as well as the improvement of oxidative stress and inflammation in cells of islets. This finding may also explain why the treatment modalities in this study had an antihyperglycemic effect. Adeva-Andany et al. (2016) reported that glucokinase activity raises the rate at which glucose can be used, while the final enzyme in the process of gluconeogenesis, the glucose-6-phosphatase enzyme, decreases the rate of new glucose production. Similarly, the increased glycogen level in the liver after the administration of blackberry juice to rats with diabetes can be associated with the increased availability of glucose-6-phosphate due to decreased Glucose-6-phosphatase activity and increased glucokinase activity. Abouzed et al. (2020) discovered that treating STZ- rats with diabetes using black mulberry fruit extract increased insulin levels compared to the normal control group, possibly due to anthocyanin content.

In diabetic rats, there was a significant decrease in CAT activity, SOD, and GPX levels compared to the normal control group, indicating lower antioxidant capacity. Conversely, the diabetic group had significantly higher levels of MDA, a marker of lipid peroxidation and oxidative stress. Oxidative stress in the diabetic group was connected to a lower antioxidant state that exacerbates the harmful impacts of free radicals (Picton 2001). Increased oxidative stress is a generally established component in the development of diabetes and its associated consequences. Increased oxidative stress may result from diabetes-related free radical generation or compromised antioxidant defenses (Maritim et al. 2003). In diabetes situations, the CAT activity and SOD values deteriorate due to unregulated production of hydrogen peroxide-induced by lipid peroxidation, protein glycation, and glucose autoxidation (Sefi et al. 2011). These findings agree with Abolfathi et al. (2012), who found that increased sensitivity to lipid peroxidation accompanied STZ-induced diabetes in rats. In diabetic rats, treatment with blackberry juice significantly reduced oxidative stress, as evidenced by increased CAT activity and levels of SOD and GPX, compared to the diabetic control group. Blackberry juice could inhibit lipid peroxidation, which is beneficial in maintaining the integrity of the transport layer fluid gradient and the actions of receptors and enzymes bound to the membrane. Maintaining the integrity of membrane lipid is crucial for preventing structural or functional problems linked to diabetes and its consequences, including atherosclerosis (Ma 2012).

Pro-inflammatory cytokines may affect the β-cell disease. It was reported that TNF-α and IL -6 were involved in β-cell dysfunction and death. At the same time, high IL-6 levels predicted developing T2D and significantly reduced insulin sensitivity in liver cells. IL-6 and TNF-α, two pro-inflammatory cytokines, affected the production of acute-phase proteins involved in developing T2D (Muhammad et al. 2016). As a result, the development of T2D is favored by increased inflammation. (Garg et al. 2012). The results showed that blackberry juice reduced hepatic TNF- and IL-6 levels in diabetic rats. These results agree with Ibitoye and Ajiboye (2018), who reported that blackberry juice was shown to reduce IL-6 and TNF-α in rats with metabolic syndrome caused by a high fructose diet.

Peripheral insulin resistance, hyperglycemia, inflammation, and gluconeogenesis in the liver increased the need for insulin production, increasing oxidative stress and ER stress in the beta cell (Papa 2012). TNF-α was shown to be directly related to ER stress and inflammation, forming a vicious cycle between the two. While suppressing NF-κB/TNF-α signaling prevented cell death in response to ER stress, it also prevented stress-induced cell death in ER. Both transcription and translation of insulin were reduced in ER-stressed β-cells (Walter and Ron 2011). ATF4 translation was increased under ER stress, despite the overall decrease in translation (Hamanaka et al. 2009). In this study, ATF4 was used as an indicator of ER stress. This study found that blackberry juice dramatically reduced the expression of ATF4 in diabetic rats, suggesting that ER stress decreased. Based on these results, insulin production and action improved. ATF4 was associated with regulating genes that protect cells from oxidative stress. Therefore, the improved antioxidant status in tissues, especially after treatment with blackberry juice, might have connections with the lower ER stress.

The liver, one of the essential endogenous organs, is severely impacted by diabetes. These findings corroborate Ghara et al. (2021), who found that STZ caused hepatocyte damage, including binucleated cells, and increased Kupffer cells, necrosis, and aberrant sinusoids. Liver damage in diabetic individuals is mainly caused by hyperglycemia-induced oxidative stress, which leads to abnormalities in glucose, protein, and lipid metabolisms (Mohamed et al. 2016). Blackberry fruit juice's antioxidant components, such as cyanidin 3-glucoside and cyanidin 3-rutinoside, have been shown to reduce oxidative stress in cells, resulting in expected structural and functional outcomes, as reported by Qin et al. (2018). Anthocyanin-rich diets were shown to reduce lipogenesis and ameliorate hepatic steatosis (Tsuda 2008). Cyanidin, a chemical found in the juice of blackberry (Pérez-Grijalva et al. 2018), was shown to drastically lower cellular lipid contents in steatotic hepatocytes loaded with lipids.

## Conclusion

Finally, the paper on rats with diabetes gave significant findings regarding the consumption of blackberry juice as it could improve lipid profile and reduce elevated levels of total cholesterol, LDL, triglyceride, glucose metabolism, and oxidative stress modulating defense mechanism. Antioxidants reduce inflammation and endoplasmic reticulum (ER) stress. Thus, it gave exciting data for choosing blackberry to be included in the functional food group.

### Limitation of study

The present study provides valuable insights into the potential therapeutic effects of blackberry juice on diabetic rats, but it is important to note its limitations. Firstly, the study was conducted on animal models and may not necessarily reflect the effects on humans. Additionally, the study only examined the effects of blackberry juice on a limited set of parameters related to diabetes and did not explore its effects on other important aspects such as lipid metabolism. Moreover, the study is limited by the lack of pancreatic tissue. Finally, the study did not investigate the potential side effects of blackberry juice consumption or its interactions with other medications. Therefore, further studies are necessary to confirm these findings and explore the full potential of blackberry juice as a therapeutic intervention for diabetes.

## Data Availability

The datasets utilized and analyzed during this investigation are available upon reasonable request from the corresponding author.
